# A Systemic Capillary Leak Syndrome (Clarkson Syndrome) in a Patient with Chronic Lymphocytic Leukemia: A Case Report in an Out-of-Hospital Setting

**DOI:** 10.1155/2016/5347039

**Published:** 2016-03-16

**Authors:** Manon Durand Bechu, Antoine Rouget, Christian Recher, Elie Azoulay, Vincent Bounes

**Affiliations:** ^1^Service d'Aide Médicale Urgente de la Haute Garonne (SAMU 31), Hôpital Universitaire de Purpan, place du Docteur Baylac, TSA 40031, 31059 Toulouse Cedex 9, France; ^2^Service d'Anesthésie-Réanimation, CHU Hôpital Rangueil, 1 avenue Jean Poulhes, TSA 50032, 31059 Toulouse Cedex 9, France; ^3^Service d'Hématologie, Pôle IUC Oncopôle, Institut Universitaire du Cancer de Toulouse, 1 avenue Irène Joliot-Curie, 31059 Toulouse Cedex 9, France; ^4^Service de Réanimation Médicale, Groupe de Recherche Respiratoire en Réanimation Onco-Hématologique, Hôpital Saint-Louis, Université Paris-Diderot, Sorbonne Paris-Cité, 1 avenue Claude Vellefaux, 75010 Paris, France

## Abstract

Systemic Capillary Leak Syndrome (SCLS) is a rare disease with poor prognosis, characterized by the occurrence of mucocutaneous and visceral edema with hypotension, hemoconcentration, and unexpected hypoalbuminemia. The disease can be idiopathic (Clarkson syndrome) or secondary to other diseases and treatments. We describe this syndrome in a prehospitalized, 63-year-old patient with chronic lymphocytic leukemia and an idiopathic form of SCLS manifesting as hypovolemic shock. Initial care is hospitalization in intensive care. In addition to etiological treatment if fluid replacement is necessary, treatment must be closely monitored for secondary overload complications. Catecholamine rather than arrhythmogenic support may be associated.

## 1. Introduction

Systemic Capillary Leak Syndrome (SCLS) is a rare disease with severe prognosis. Capillary endothelium hyperpermeability causes extravascular leakage of plasma into the interstitial area, explaining the double symptoms of this disease: mucocutaneous and visceral edema, as well as hypovolemic shock with hemoconcentration with paradoxical hypoalbuminemia. The disease can be idiopathic (Clarkson syndrome) or secondary to other diseases and treatments [1]. Initially, the main risk is acute pulmonary edema and compartment syndrome, aggravated by massive vascular filling [1]. This study describes the syndrome in a prehospitalized patient.

## 2. Case Presentation

A 63-year-old man presented with two days' digestive symptoms: nausea, vomiting, diarrhea, and abdominal pain with fever and asthenia. The patient had a history of hypertension (treated with amlodipine), chronic lymphocytic leukemia stage A with a monoclonal gamma peak, and septic shock in 2012. Six month earlier, a routine blood sample analysis showed no abnormality with the notable exception of a peak of gammaglobulins (16,4 g/L). At his home, the paramedical team found high blood pressure and oxygen saturation and a heart rate of 102 per minute in a pale patient, sweating and asthenic, and no neurological disorder. He had abdominal pain without tenderness, nausea, and hypothermia at 33.8°C. He showed no chest pain, no signs of heart failure, and no radial pulse, and his veins of the lower limbs and abdomen were apparent. He had a pulmonary surface polypnea at 34 per minute and cyanosis of the extremities (SpO_2_ 84%), but normal auscultation. HemoCue was at 23.6 g/dL and glucose was at 1.7 g/L. Proteinuria was negative on a urinal quick test, the protein was 43 g/L, and glomerular renal filtration was found at 30 mL/min. Albumin was at 24.7 g/L and all blood immunoglobulins were low. Concerning liver laboratory findings, only gamma GT was 3 times higher than normal. The electrocardiogram revealed a sinus rhythm at 100 per minute with a narrow QRS without conduction disturbance or repolarization.

After 750 mL of vascular filling and a continuous unstable hemodynamic status, norepinephrine was introduced. Orotracheal intubation through ventilatory depletion after rapid sequence induction was then carried out.

A thoracoabdominal pelvic CT scan did not find any abnormality or visceral edema. Biological results revealed hyperhemoglobin at 23 g/dL albumin associated with paradoxical hypoalbuminemia at 20 g/L. Hematological results then evoked a diagnosis of SCLS. Intravenous immunoglobulin therapy CLAIRYG® was established.

Within hours, the clinical condition deteriorated hemodynamically requiring new massive filling and an increase of catecholamine doses. In this hypoxemic and vasoplegic context with multiple organ failure and persistent acidosis, the patient presented with refractory cardiac arrest and died minutes later. All bacteriological samples were sterile.

## 3. Discussion

Systemic Capillary Leak Syndrome was first described in 1960 [2]. Analysis of cases published since shows that the average age of onset of symptoms is about 45 years without sex predominance (M/F = 1.2) [1, 3]. Subjects are mostly Caucasians [4], and rare pediatric forms have been reported [1, 4–6]. Secondary forms are primarily associated with hematological malignancies, viral infections in particular, and certain medications including blood cancer [1]. Diagnosis of idiopathic SCLS is evoked without predisposing circumstances, particularly since there is a monoclonal immunoglobulin in the majority of cases [1] with a recurrent seizure characteristic. Concerning this case, a nephrotic syndrome was evoked but not compatible with the negative proteinuria, and we excluded a transition into some different hematologic malignoma, considering the normality of the liver laboratory findings.

The typical clinical presentation consists of three phases: the prodromal phase until the 48th hour with weight gain, fatigue, faintness, low-grade fever, and head and neck or gastrointestinal symptoms related to mucosal edema. The status phase includes weight gain, oedema in the lungs, oliguria, hypotension up to and including shock, and preserved consciousness. The pressure invasive monitoring and cardiovascular rates confirm the diagnosis of hypovolemic shock [7–9]. During recovery beginning between the 5th and 7th day, the patient feels a sense of “feeling better” with normalization of blood pressure, polyuria, disappearance of oedema, and weight loss [1].

Laboratory abnormalities most often encountered are those presented by this patient with hemoconcentration, hyposerum protein and hypoalbumin, decreased TP leakage factor 5, acute renal failure, and rhabdomyolysis [1, 4, 10]. Complications are caused by the transfer of the vascular sector into the interstitial area with acute pulmonary edema and rhabdomyolysis, particularly since, in this case, vascular filling would have been massive and rapid [1].

The therapeutic management is common to idiopathic or secondary SCLS. The patient should be transferred to intensive care and fluid resuscitation must be given with caution with isotonic NaCl and guided by diuresis. Vasoactive amines are recommended in case of early signs of hypoperfusion, if possible with norepinephrine because it is less arrhythmogenic and if necessary extrarenal purging in case of acute renal failure [1, 10, 11].

In cases of idiopathic SCLS, long-term treatments have been proposed by some authors, but without formally being proven effective. They target antihistamines and beta 2 agonists with terbutaline and theophylline [1, 4, 10–13]. More recently in a European register, analysis of patients treated with immunoglobulin intravenously at a dose of 2 g/kg per month showed a decrease in the frequency of occurrence and severity of seizures in treated patients. Curative proof is still low at this time [10, 14].

## References

[1] Duron L., Delestre F., Amoura Z., Arnaud L. (2015). Syndrome de fuite capillaire idiopathique et formes secondaires: une revue systématique de la littérature. *La Revue de Médecine Interne*.

[2] Clarkson B., Thompson D., Horwith M., Luckey E. H. (1960). Cyclical edema and shock due to increased capillary permeability. *The American Journal of Medicine*.

[3] Druey K. M., Greipp P. R. (2010). Narrative review: the systemic capillary leak syndrome. *Annals of Internal Medicine*.

[4] Gousseff M., Amoura Z. (2009). Idiopathic capillary leak syndrome. *Revue de Medecine Interne*.

[5] Kerketta J., Lodh M., Mandal K. (2015). Clarkson disease—systemic capillary leak syndrome in a 6-year-old girl: case report. *Paediatrics and International Child Health*.

[6] Hsu P., Xie Z., Frith K. (2015). Idiopathic systemic capillary leak syndrome in children. *Pediatrics*.

[7] George C., Regnier B., Le Gall J. R., Gastinne H., Carlet J., Rapin M. (1978). Hypovolaemic shock with oedema due to increased capillary permeability. *Intensive Care Medicine*.

[8] Jacox R. F., Waterhouse C., Tobin R. (1973). Periodic disease associated with muscle destruction. *The American Journal of Medicine*.

[9] Noirot A., Delacour J. L., Floriot C., Kramarz P., Wagshal G., Daoudal P. (1991). Choc hypovolémique par hyperperméabilité capillaire. Un cas sans dysglobulinémie monoclonale. *La Presse Médicale*.

[10] Gousseff M., Arnaud L., Lambert M. (2011). The systemic capillary leak syndrome: a case series of 28 patients from a European registry. *Annals of Internal Medicine*.

[11] Amoura Z., Papo T., Ninet J. (1997). Systemic capillary leak syndrome: report on 13 patients with special focus on course and treatment. *American Journal of Medicine*.

[12] Droder R. M., Kyle R. A., Greipp P. R. (1992). Control of systemic capillary leak syndrome with aminophylline and terbutaline. *American Journal of Medicine*.

[13] Kapoor P., Greipp P. T., Schaefer E. W. (2010). Idiopathic systemic capillary leak syndrome (Clarkson's disease): the Mayo Clinic experience original article. *Mayo Clinic Proceedings*.

[14] Lambert M., Launay D., Hachulla E. (2008). High-dose intravenous immunoglobulins dramatically reverse systemic capillary leak syndrome. *Critical Care Medicine*.

